# Transvaginal Ultrasound for Endometriosis: Learning From a Decade of Diagnostic Challenges Through a Retrospective Observational Cohort Study

**DOI:** 10.1111/ajo.70183

**Published:** 2026-09-29

**Authors:** Candice Dry, Charlotte Moss, Elizabeth Nathan, Michelle K. Pedretti, Glen Lo, Phillipa Robertson, Bernadette Mcelhinney, Emma R. Allanson, Jan E. Dickinson

**Affiliations:** ^1^ King Edward Memorial Hospital Women and Newborn Health Service Perth Australia; ^2^ Division of Obstetrics and Gynaecology The University of Western Australia Perth Australia

**Keywords:** #ENZIAN classification, deep endometriosis, diagnostic accuracy, endometriosis, transvaginal ultrasound

## Abstract

**Background:**

Endometriosis is a chronic, inflammatory condition associated with pelvic pain, subfertility, and reduced quality of life. Advances in enhanced transvaginal ultrasound (eTVUS), including systematic compartment‐based scanning protocols and improved operator training, have increased diagnostic accuracy, particularly for deep endometriosis.

**Aims:**

To evaluate and critically reflect on the diagnostic performance of eTVUS during its implementation prior to the adoption of standardised scanning protocols. Using the #ENZIAN classification system, the diagnostic performance of eTVUS was assessed by comparing ultrasound findings with laparoscopic visualisation as the reference standard.

**Methods:**

A retrospective observational study of patients with suspected endometriosis who underwent preoperative eTVUS and subsequent laparoscopy between January 2015 and January 2024 at a single tertiary hospital. Ultrasound and surgical findings were mapped to #ENZIAN compartments and grades. Agreement and diagnostic metrics were calculated using laparoscopy as the reference standard.

**Results:**

Among 221 patients with paired ultrasound and laparoscopy, adnexal endometriomas were detected with a sensitivity of 81.4% and specificity of 84.1%. Tubo‐ovarian complex disease demonstrated a sensitivity of 72.9% and specificity of 68.8%. Sensitivity for deep endometriosis was lower, with specificity for detection depending on anatomical location varying from 83%–95%. Bladder involvement had a PPV of 83.3% and NPV of 94.8%; ureteric disease had a PPV of 0% and NPV of ~100%. Peritoneal disease was rarely identified.

**Conclusion:**

eTVUS demonstrated high diagnostic accuracy for ovarian endometriomas, moderate performance for bowel involvement, and low sensitivity for peritoneal disease.

## 1 Introduction

The Royal Australian and New Zealand College of Obstetricians and Gynaecologists ‘Living Evidence Guideline: Endometriosis’ defines endometriosis as a chronic gynaecological condition characterised by endometrial‐like tissue located outside the uterus, typically accompanied by an inflammatory process. It may be a debilitating disease and is often associated with pelvic pain, subfertility, and substantially reduced quality of life [1]. The International Deep Endometriosis Analysis (IDEA) group consensus subdivides endometriosis according to its imaging phenotypes, which include superficial peritoneal endometriosis (SPE), ovarian endometrioma (OE), and deep endometriosis (DE) involving the uterosacral ligaments, rectovaginal septum, bowel, bladder and the parametrium [2]. Contemporary terminology favours ‘deep endometriosis’ over ‘deep infiltrating endometriosis’, reflecting a shift from the traditional ≥ 5 mm infiltration criterion to a definition based on invasion of fibromuscular or organ structures, aligning with the IDEA framework [2].

Endometriosis affects approximately 1 in 7 Australian women by midlife, with prevalence estimates ranging from 10%–14%, depending on age and diagnostic criteria [3]. Diagnosis remains challenging; however, due to the heterogeneous presentation and anatomical variability, which can contribute to delayed recognition and treatment [4, 5].

Historically, transvaginal ultrasound (TVUS) was considered inadequate for detecting endometriosis, particularly DE, leading to reliance on laparoscopy and an average diagnostic delay from time of presentation with symptoms of 6.6 years (range 1.5–11.3 years) [6]. This misconception, combined with the absence of standardised protocols and limited operator training, resulted in frequent diagnostic errors and over‐reporting of ‘normal’ imaging findings in patients with advanced disease [5].

To address the historical limitations of routine imaging, enhanced transvaginal ultrasound (eTVUS) was developed as a structured, compartment‐based protocol based on the International Deep Endometriosis Analysis (IDEA) recommendations. Unlike traditional ultrasound, which primarily focuses on the uterus and ovaries, eTVUS follows a systematic assessment of the pelvis, including rectovaginal septum, parametria, uterosacral ligaments, and pelvic large bowel.

Over the past decade, advances including the integration of dynamic manoeuvres (e.g., organ mobility assessment) and structured reporting frameworks have significantly improved eTVUS diagnostic performance [7]. Diagnostic performance is further improved when ultrasound findings are preoperatively reviewed by a multidisciplinary team, incorporating input from gynaecologists, radiologists and sonologists, minimally invasive gynaecologic surgeons, and colorectal or urological specialists (when relevant). This collaborative approach facilitates interpretation and the resolution of potential discordance between eTVUS and magnetic resonance imaging (MRI), reducing false negative and false positive findings, and optimising patient care [7, 8]. Diagnostic challenges remain, however, particularly for superficial endometriotic lesions (i.e., peritoneal disease), tubal disease and uterosacral ligament lesions, where sonographic diagnostic accuracy is still low [9]. Transvaginal ultrasound is now widely regarded as a reliable first‐line imaging modality for suspected endometriosis [7, 10]. While laparoscopy remains the reference standard by offering direct visualisation and subsequent histological confirmation of lesions, it is no longer recommended as a first‐line investigation [1]. Thus, preoperative imaging plays two key roles: Establishing a non‐invasive diagnosis through high‐quality eTVUS and providing detailed disease mapping to guide surgical planning [1, 2, 7].

The #ENZIAN classification system, named after the alpine gentian flower (“Enzian” in German), was originally developed as a surgical framework for describing endometriosis and documenting disease extent at surgery [11]. The updated #ENZIAN classification incorporates peritoneal, ovarian, tubo‐ovarian, deep, adenomyotic, and organ‐specific disease within a unified compartment‐based system applicable to both imaging and surgical settings [11, 12]. Adenomyosis is included because it commonly coexists with endometriosis and may represent a related disease phenotype [13]. This facilitates standardised disease description, multidisciplinary communication, and direct comparison between preoperative imaging and operative findings [11, 12]. In the present study, historical ultrasound and operative reports were retrospectively mapped to #ENZIAN categories to enable evaluation of imaging‐surgical concordance. Detailed classification procedures are described in the Methods section.

In this study, #ENZIAN classification was applied retrospectively as a structured framework to harmonise imaging and surgical findings. While the IDEA consensus provides a standardised protocol for the systematic acquisition and reporting of endometriosis on ultrasound, its widespread implementation occurred later in the study period and was variably adopted in earlier examinations within this cohort. As such, historical ultrasound and operative reports were not generated using a uniform reporting system. The #ENZIAN classification was therefore selected as it provides a comprehensive, compartment‐based structure encompassing peritoneal, ovarian, tubo‐ovarian, deep, and organ‐specific disease, and is applicable across both imaging and surgical settings. This allowed consistent mapping of free‐text reports into a common anatomical framework, facilitating direct comparison between preoperative eTVUS findings and laparoscopic disease distribution. This approach aligns with the primary aim of evaluating imaging: Surgical concordance within a real‐world cohort during the early implementation of structured endometriosis ultrasound protocols.

This study aimed to evaluate the diagnostic accuracy of eTVUS in detecting endometriosis over 10 years at a single tertiary obstetric and gynaecology hospital by comparing ultrasound findings with laparoscopic results. By retrospectively applying the #ENZIAN classification, we sought to evaluate historical diagnostic performance, identify strengths and limitations of ultrasound assessment, and derive lessons to inform future practice.

## 2 Methods

### 2.1 Study Design and Setting

This retrospective, observational study was conducted at King Edward Memorial Hospital, a statewide tertiary referral centre for women's reproductive health, encompassing the time period from 1 January 2015 to 1 January 2024. The cohort reflects the evolution of enhanced transvaginal ultrasound (eTVUS) within the imaging department, capturing patients assessed during the early implementation phase of eTVUS in a hospital‐based ultrasound service.

### 2.2 Operator Expertise

Ultrasound examinations were performed within a tertiary referral imaging service by a combination of experienced sonographers, sonologists, and radiologists with varying levels of expertise in gynaecological imaging and endometriosis assessment. Over the study period, operator experience evolved alongside the implementation of enhanced transvaginal ultrasound (eTVUS), with increasing familiarity in assessing deep endometriosis and performing compartment‐based evaluation. As this study reflects an early phase of protocol development, variability in operator skill, confidence, and pattern recognition, particularly for subtle or non‐cystic disease, was present and likely influenced diagnostic performance across anatomical compartments.

Laparoscopic procedures were performed in a tertiary referral setting by a combination of Advanced Gynaecological Endoscopic Society (AGES) endometriosis surgeons or by an AGES fellow in training under the supervision of one or two AGES‐trained surgeons. Consultant presence and involvement were guided by the severity of the disease. The majority of the cases were completed by the fellow with the consultant either scrubbed in to assist or directly observing. At times, however, the consultant would become the primary operator pending case complexity.

### 2.3 Case Identification

Eligible cases were first identified through a Radiology Information System (RIS) search for any report using the keywords “deep infiltrating endometriosis (DIE)” within the imaging indication or report text. The unique medical record number (UMRN), associated with each imaging entry, was then cross‐linked to the hospital theatre management system to identify patients who subsequently underwent laparoscopy for suspected endometriosis during the study period. For those with multiple occasions of pre‐operative imaging, the most recent eTVUS prior to surgery was designated as the index imaging study. Patients were included if both a pre‐operative eTVUS performed for suspected endometriosis and corresponding documented operative findings were available.

Eligible cases were included if they fulfilled the following criteria:

1. Preoperative eTVUS report.
2. Documented operative findings from laparoscopy.
3. Clinical management for suspected or confirmed endometriosis during the study period.

The case selection process is summarised in Figure 1.

**FIGURE 1:**
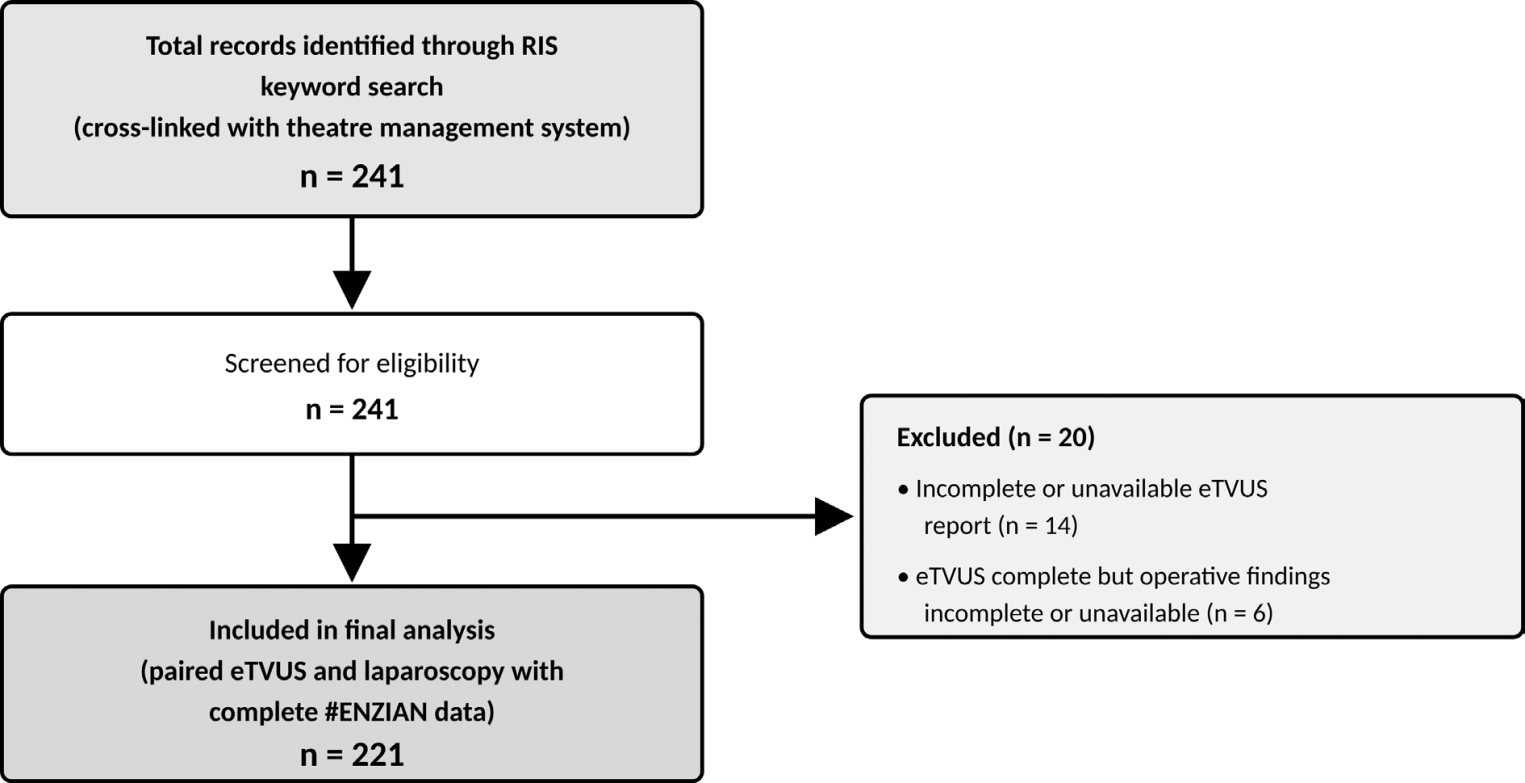
Participant flow diagram. Records were identified in a RIS keyword search for “deep infiltrating endometriosis” and cross‐linked to the hospital theatre management system. Of 241 records screened for eligibility, 20 were excluded (incomplete or unavailable eTVUS report, *n* = 14; eTVUS complete but operative findings incomplete or unavailable, *n* = 6), leaving 221 with paired eTVUS and laparoscopy and complete #ENZIAN data included in the final analysis. eTVUS = enhanced transvaginal ultrasound; RIS = Radiology Information System.

Patients were excluded if [1]: no laparoscopy was performed or identified within the study period [2]; operative findings were incomplete or unavailable for extraction [10]; the eTVUS was not performed for suspected endometriosis (i.e., performed solely for an unrelated indication). Where a patient had undergone multiple preoperative eTVUS examinations, only the most recent examination prior to laparoscopy was retained as the index study, with earlier examinations excluded from analysis.

The #ENZIAN classification [10] was used to categorise pre‐operative eTVUS findings and the corresponding laparoscopic findings for each case. Given the retrospective nature of the study and the absence of standardised #ENZIAN reporting during much of the study period, historical ultrasound and operative reports were retrospectively mapped to #ENZIAN compartments and severity grades. This approach was selected because #ENZIAN provides a common anatomical classification framework applicable to both imaging and surgical findings, enabling direct comparison of disease distribution and severity between modalities. As many examinations pre‐dated widespread implementation of IDEA‐based acquisition and reporting protocols, report structure and terminology were variable. Retrospective application of #ENZIAN therefore provided a practical method for harmonising free‐text reports and assessing imaging‐surgical concordance across the cohort. #ENZIAN scores were retrospectively assigned by translating the descriptive content of the ultrasound report and the operative report into the #ENZIAN compartments and severity grades.

Due to the retrospective structure of historical ultrasound and operative reports, complete application of all #ENZIAN elements (e.g., F‐categories) was not always possible; however, available #ENZIAN components were applied consistently across the dataset. Classification was initially performed by an assigned clinical investigator, with any uncertain or complex cases reviewed by either the clinical principal investigator (CPI) or the primary author to ensure accuracy and minimise bias. This classification provides a morphologically descriptive classification of endometriosis, including presence of peritoneal lesions, ovarian endometriomas, tubal involvement, and deep endometriosis.

Disease location was mapped to the three primary #ENZIAN compartments:

- Compartment A: Vagina and rectovaginal septum.
- Compartment B: Uterosacral ligaments, cardinal ligaments, and pelvic sidewall.
- Compartment C: Rectum and sigmoid colon.

The #ENZIAN severity grading was assigned according to lesion size within each compartment, with Grade 1 representing lesions < 1 cm, Grade 2 representing lesions 1–3 cm, and Grade 3 representing lesions > 3 cm. Tubal condition was documented using the ENZIAN T‐descriptor, characterising the fallopian tubes as normal (T0), affected by adhesions (T1), distorted or fixed (T2), or demonstrating hydrosalpinx or severe tubal pathology (T3). Extra‐pelvic involvement and organ‐specific disease were recorded separately using the #ENZIAN F‐categories, including FA (adenomyosis), FB (bladder involvement), FU (ureteric involvement), FI (bowel disease), and FO (i.e., diaphragm, lung, or nerve) [10].

### 2.4 Data Collection

Data collected from the pre‐operative transvaginal ultrasound (eTVUS) reports included the lesion location and type, which were converted to #ENZIAN classification, pouch of Douglas (POD) status, and the overall ultrasound impression. Operative findings (including the surgeon's overall diagnostic impression) were similarly recorded from the laparoscopy reports. When required, free‐text descriptions from imaging or operative reports were systematically harmonised into #ENZIAN categories using pre‐specified coding rules [10] to minimise misclassification and ensure consistency between imaging and surgical datasets.

Study data were collected and managed using REDCap (Research Electronic Data Capture), a secure web‐based application commonly used for research data entry and management [14, 15].

### 2.5 Confidentiality and Ethics

Ethics approval for this study was obtained through the Child and Adolescent Health Service Human Research Ethics Committee (Approval No: EC00268). The study was classified as low risk under the National Statement on Ethical Conduct in Human Research [16].

### 2.6 Statistical Analysis

Descriptive statistics were presented as mean ± standard deviation (SD) for normally distributed continuous variables, and median (interquartile range [IQR]) for non‐parametric data. Categorical variables were summarised as counts and percentages.

Agreement between ultrasound and laparoscopic #ENZIAN severity scores was assessed using Cohen's weighted kappa with quadratic weights. These agreement measures reflect concordance between imaging findings and the surgical reference standard and should not be interpreted as inter‐observer reliability between operators.

For diagnostic performance, #ENZIAN scores were dichotomised into positive/negative findings relative to laparoscopy (used as the reference standard). Sensitivity, specificity, accuracy, PPV, and NPV were calculated with 95% confidence intervals (CI). Kappa was interpreted as: < 0.20 poor, 0.21–0.40 fair, 0.41–0.60 moderate, 0.61–0.80 substantial, > 0.80 almost perfect agreement.

Statistical analyses were performed by a hospital biostatistician using Microsoft Excel and IBM SPSS Statistics Version 29.0 (IBM Corp., Armonk, NY) in consultation with the study investigators.

## 3 Results

A total of 221 patients were identified with both ultrasound and laparoscopic findings available and met the study criteria for analysis. The mean age of the patients was 38.4 years, with more than half being nulliparous. The patient demographic characteristics are summarised in Table 1. The most common presenting symptoms in patients undergoing ultrasound imaging were pelvic pain (167/221, 75.6%) and dysmenorrhoea (143/221, 64.7%). Hormonal therapies were used by 144/221 (59.8%) of patients, and 161/221 (72%) in this cohort had undergone prior surgery for endometriosis (Table 1).

**TABLE 1:** Demographic characteristics.

Characteristic	*N* (%)
Age (years) (mean, SD)^a^	38.4 (7.9)
BMI (kg/m^2^) (median, IQR, R) (*n* = 201)	25.5 (22.8–30.7, 17.8–54.9)
Parity	
0	127 (57.5)
1	46 (20.8)
2	28 (12.7)
3 or more	12 (5.4)
Missing	8 (3.6)
Diagnosis	
Endometriosis	205 (92.8)
Adenomyosis	11 (5.0)
Bowel DE	26 (11.8)
Presenting symptoms	
Pelvic pain	167 (75.6)
Dysmenorrhoea	143 (64.7)
Dyspareunia	75 (33.9)
Dyschezia	71 (32.1)
Menorrhagia	64 (29.0)
Infertility	53 (24.0)
Dysuria	18 (8.1)
Previous endometriosis surgery	161 (72.9)
Hormonal treatment	
Progesterone IUD^b^	48 (19.9)
Combined oral contraceptive pill	38 (15.8)
Dienogest	24 (10.0)
Oral progesterone	16 (6.6)
Other^c^	18 (7.5)

^a^ Age data were available for 218 participants.

^b^ Intrauterine device.

^c^ Other hormone includes Zoladex (*n* = 8), Implanon (*n* = 4) and Depo‐Provera (*n* = 6).

### 3.1 Agreement Between Ultrasound and Laparoscopic Findings

On direct visualisation at laparoscopy, endometriosis was documented in 160 patients (72.4%). Among compartments with available documentation, superficial peritoneal disease was noted in 35/139 (25.2%). Ovarian endometriomas were present in 68/194 (35.1%) left‐sided and 61/192 (31.8%) right‐sided. Deep endometriosis was most frequently recorded in compartment B; 87/184 (47.3%) left and 82/182 (45.1%) right, followed by compartment A, 52/183 (28.4%) and compartment C, 33/185 (17.8%). Bladder and small‐bowel involvement were less common (16/202 (7.9%) and 16/212 (7.5%), respectively), with ureteric disease in 14/220 (6.4%) and Pouch of Douglas obliteration in 26/64 (40.6%). Variable denominators reflect documentation availability per site.

Agreement between ultrasound and laparoscopic #ENZIAN severity scores varied by anatomical site (Table 2). Overall percentage agreement ranged from 49.5% to 74.6%, with Cohen's weighted kappa values indicating poor to substantial agreement depending on location.

**TABLE 2:** Percentage agreement between ultrasound scores and laparoscopic surgery (cells shaded in grey). cohen's weighted kappa estimation used quadratic weights.

Ultrasound	Laparoscopy surgery				Overall agreement	Weighted kappa (95% CI)
	None visible	< 3 cm	3–7 cm	> 7 cm		
Peritoneum						
None visible	100 (96.2)	31 (96.9)	1 (50.0)	0 (0.0)	72.7%	0.24 (0.00–0.54)
< 3 cm	2 (1.9)	0 (0.0)	0 (0.0)	0 (0.0)		
3–7 cm	1 (1.0)	1 (3.1)	1 (50.0)	1 (100)		
> 7 cm	1 (1.0)	0 (0.0)	0 (0.0)	0 (0.0)		
Left ovary						
None visible	106 (84.1)	8 (25.0)	3 (14.3)	1 (6.7)	72.7%	0.73 (0.64–0.82)
< 3 cm	13 (10.3)	12 (37.5)	2 (9.5)	1 (6.7)		
3–7 cm	7 (5.6)	9 (28.1)	13 (61.9)	3 (20.0)		
> 7 cm	0 (0.0)	3 (9.4)	3 (14.3)	10 (66.7)		
Right ovary						
None visible	110 (84.0)	8 (33.3)	4 (17.4)	0 (0.0)	70.3%	0.71 (61–0.80)
< 3 cm	9 (6.9)	5 (20.8)	5 (21.7)	0 (0.0)		
3–7 cm	11 (8.4)	9 (37.5)	9 (39.1)	3 (21.4)		
> 7 cm	1 (0.8)	2 (8.3)	5 (21.7)	11 (78.6)		
Left tubal ovarian	**T0**	**T1**	**T2**	**T3**		
T0	63 (65.6)	15 (55.6)	2 (9.1)	11 (19.3)	49.5%	0.43 (0.31–0.56)
T1	8 (8.3)	5 (18.5)	2 (9.1)	7 (12.3)		
T2	15 (15.6)	5 (18.5)	14 (63.6)	21 (36.8)		
T3	10 (10.4)	2 (7.4)	4 (18.2)	18 (31.6)		
Right tubal ovarian						
T0	67 (72.0)	17 (51.5)	3 (13.0)	11 (20.0)	48.5%	0.45 (0.34–0.57)
T1	6 (6.5)	4 (12.1)	2 (8.7)	5 (9.1)		
T2	12 (12.9)	5 (15.2)	13 (56.5)	24 (43.6)		
T3	8 (8.6)	7 (21.2)	5 (21.7)	15 (27.3)		
Compartment A	**None visible**	**< 1 cm**	**1**–**3 cm**	**> 3 cm**		
None visible	125 (95.4)	34 (72.3)	2 (50.0)	0 (0.0)	74.3%	0.47 (0.29–0.65)
< 1 cm	6 (4.6)	8 (17.0)	0 (0.0)	0 (0.0)		
1–3 cm	0 (0.0)	2 (4.3)	2 (50.0)	0 (0.0)		
> 3 cm	0 (0.0)	3 (6.4)	0 (0.0)	1 (100.0)		
Left Compartment B						
None visible	83 (85.6)	56 (69.1)	1 (20.0)	9 (0.0)	59.8%	0.28 (0.11–0.45)
< 1 cm	10 (10.3)	24 (29.6)	0 (0.0)	1 (100.0)		
1–3 cm	3 (3.1)	1 (1.2)	3 (60.0)	0 (0.0)		
> 3 cm	1 (1.0)	0 (0.0)	1 (20.0)	0 (0.0)		
Right Compartment B						
None visible	83 (93.0)	53 (72.6)	4 (57.1)	0 (0.0)	56.0%	0.27 (0.08–0.45)
< 1 cm	16 (16.0)	17 (23.3)	2 (28.6)	1 (50.0)		
1–3 cm	1 (1.0)	2 (2.7)	1 (14.3)	0 (0.0)		
> 3 cm	0 (0.0)	1 (1.4)	0 (0.0)	1 (50.0)		
Compartment C						
None visible	132 (86.8)	15 (57.7)	1 (25.0)	1 (33.3)	74.6%	0.40 (0.20–0.59)
< 1 cm	8 (5.3)	2 (7.7)	0 (0.0)	0 (0.0)		
1–3 cm	9 (5.9)	7 (26.9)	2 (50.0)	0 (0.0)		
> 3 cm	3 (2.0)	2 (7.7)	1 (25.0)	2 (66.7)		

*Note:* Cohen's weighted kappa estimation used quadratic weights.

The highest levels of agreement were observed for ovarian endometriomas, with substantial agreement for both the left ovary (*κ* = 0.73, 95% CI 0.64–0.82) and the right ovary (*κ* = 0.71, 95% CI 0.61–0.80), indicating good concordance between ultrasound size estimation and laparoscopic findings. By contrast, agreement was lowest for superficial peritoneal disease, which demonstrated poor agreement (*κ* = 0.24, 95% CI 0.00–0.54).

For deep endometriosis, agreement was site‐dependent. Moderate agreement was observed for compartment C (rectum/sigmoid colon) (*κ* = 0.40, 95% CI 0.20–0.59). The agreement for the uterosacral ligament compartments (compartment B) was fair; consistently lower (*κ*≈0.27–0.28).

### 3.2 Diagnostic Performance

Ultrasound demonstrated variable sensitivity and specificity across compartments and organ systems (Table 3):

- Ovarian endometriomas: Sensitivity 80%–82%, specificity 84%, accuracy > 82%.
- Tubal‐ovarian conditions: Sensitivity ~72%–74%, specificity ~65%–72%.
- Compartment A (rectovaginal septum): Sensitivity 30.8%, specificity 95.4%.
- Compartment B (uterosacral ligaments): Sensitivity 30%–34%, specificity 83%–85%.
- Compartment C (rectum/sigmoid): Sensitivity 48.5%, specificity 86.8%.
- Bladder and ureteric disease: Sensitivity very low (0%–31%), specificity > 99%.
- Overall endometriosis diagnosis: Sensitivity 85%, specificity 65.6%, accuracy 79.6%.

**TABLE 3:** Diagnostic performance of the eTVUS across #ENZIAN‐defined disease categories (including superficial, deep, adnexal, and organ‐specific endometriosis) compared with laparoscopic findings.

	*N*	Lap	US +ve	US –ve	Sensitivity (%) 95% CI	Specificity (%) 95% CI	Accuracy (%) 95% CI	PPV (%) 95% CI	NPV (%) 95% CI
Peritoneum	139								
+ve		35	3	32	8.6 (1.8–23.1)	96.2 (90.4–98.9)	74.1 (66.0–81.2)	42.9 (15.0–76.1)	75.8 (73.7–77.7)
–ve		104	4	100					
Ovary (L)	194								
+ve		68	56	12	82.4 (71.2–90.5)	84.1 (76.6–90.0)	83.5 (77.5–88.4)	73.7 (64.9–80.9)	89.8 (84.0–93.7)
–ve		126	20	106					
Ovary (R)	192								
+ve		61	49	12	80.3 (68.2–89.4)	84.0 (76.6–89.8)	82.8 (76.7–87.9)	70.0 (60.7–77.9)	90.2 (84.6–93.9)
–ve		131	21	110					
Tubal ovarian condition (L)	202								
+ve		106	78	28	73.6 (64.1–81.7)	65.6 (55.2–75.0)	69.8 (63.0–76.1)	70.3 (63.7–76.1)	69.2 (61.3–76.1)
–ve		96	33	63					
Tubal ovarian condition (R)	204								
+ve		111	80	31	72.1 (62.8–80.2)	72.0 (61.8–80.9)	72.1 (65.4–78.1)	75.5 (68.5–81.3)	68.4 (61.0–74.9)
–ve		93	26	67					
Compartment A: vagina, recto‐vaginal septum	183								
+ve		52	16	36	30.8 (18.7–45.1)	95.4 (90.3–98.3)	77.1 (70.3–82.9)	72.3 (52.5–86.6)	77.6 (74.3–80.7)
–ve		131	6	125					
Compartment B (L): uterosacral ligaments to the pelvic wall	184								
+ve		87	30	57	34.5 (24.6–45.4)	85.6 (77.0–91.9)	61.4 (54.0–68.5)	68.2 (54.9–79.0)	59.3 (55.1–63.4)
–ve		97	14	83					
Compartment B (R): uterosacral ligaments to the pelvic wall	182								
+ve		82	25	57	30.5 (20.8–41.6)	83.0 (74.2–89.8)	59.3 (51.8–66.6)	59.5 (46.1–71.7)	59.3 (51.8–66.6)
–ve		100	17	83					
Compartment C: rectum and sigmoid colon	185								
+ve		33	16	17	48.5 (30.8–66.5)	86.8 (80.4–91.8)	80.0 (73.5–85.5)	44.4 (31.8–57.8)	88.6 (84.7–91.6)
–ve		152	20	132					
Adenomyosis	217								
+ve		29	17	12	58.6 (38.9–76.5)	82.5 (76.2–87.6)	79.3 (73.3–84.5)	34.0 (25.0–44.3)	92.8 (89.3–95.2)
–ve		188	33	155					
Bladder	202								
+ve		16	5	11	31.3 (11.0–58.7)	99.5 (97.3–99.9)	94.5 (90.6–97.1)	83.3 (38.3–97.6)	94.8 (92.9–96.2)
–ve		202	1	201					
Intestinum	212								
+ve		16	7	9	43.8 (19.8–70.1)	89.8 (84.7–93.7)	86.3 (81.0–90.6)	25.9 (14.9–41.2)	95.1 (92.7–96.8)
–ve		196	20	176					
Ureter	220								
+ve		14	0	14	0.0 (0.0–23.2)	99.5 (97.3–99.9)	93.2 (89.0–96.1)	0.0	1.0
–ve		206	1	205					
PoD_condition	64								
+ve		26	20	6	76.9 (56.5–91.0)	86.8 (71.9–95.6)	82.8 (71.3–91.1)	80.0 (63.3–90.3)	84.6 (73.0–91.8)
–ve		38	5	33					
Endometriosis	221								
+ve		160	136	24	85.0 (78.5–90.2)	65.6 (52.3–77.3)	79.6 (73.7–84.7)	86.6 (82.0–90.2)	62.5 (52.5–71.6)
–ve		61	21	40					

*Note:* Diagnostic performance measures are presented for all disease subtypes classified within the #ENZIAN framework. These include Superficial peritoneal disease (peritoneal compartment), Deep endometriosis (compartments A, B, and C), Ovarian endometriomas, Tubo‐ovarian disease, Adenomyosis, and organ‐specific disease (including bladder, ureter, and bowel involvement). Sensitivity, specificity, accuracy, positive predictive value (PPV), and negative predictive value (NPV) are calculated using laparoscopic findings as the reference standard.

## 4 Discussion

This review evaluated the diagnostic accuracy of eTVUS for detecting endometriosis compared with laparoscopic findings, drawing lessons from historical limitations to inform current and future practice. It highlights key insights regarding the strengths and weaknesses of ultrasound assessment as reflected in this dataset and underscores the importance of structured protocols and consistent reporting when managing this complex multisystem disease.

The findings from this study indicate that ultrasound can identify endometriosis when key anatomical regions are systematically interrogated, and examinations are performed using a structured approach. Variation in diagnostic performance across compartments may reflect operator expertise, limited understanding of complex disease patterns, and inconsistent interrogation of relevant anatomical sites.

Accuracy for ovarian endometriomas was high, consistent with the long‐standing routine evaluation of the ovaries during pelvic ultrasound [5, 9]. Moderate performance for bowel involvement likely reflects evolving practice within this timeline, with the assumption that cases were accurately diagnosed when bowel disease was suspected, and the correct anatomical areas were reported by an experienced sonologist or radiologist. Conversely, sensitivity for peritoneal disease was low, reflecting the inherent limitations of ultrasound in visualising small peritoneal lesions, particularly in the absence of free fluid in the pelvis [5]. This is, however, an area of rapid evolution, with recent evidence suggesting that the presence of peritoneal free fluid can act as a natural acoustic window, significantly enhancing the visualisation of superficial lesions [4]. This reinforces the need for comprehensive compartmental interrogation, including assessment of organ mobility and targeted site tenderness. Low sensitivity for uterosacral lesions is not unexpected in this cohort, again likely to reflect the historic lack of systematic interrogation of the uterosacral ligaments and limited recognition of their clinical relevance in endometriosis and surgical planning [5, 7]. Taken together, these findings suggest that while ultrasound demonstrates reasonable concordance with laparoscopy for bowel deep endometriosis, agreement is more limited for disease involving the uterosacral ligaments and superficial peritoneum. Diagnostic performance in this cohort could be interpreted as reflecting early implementation of eTVUS rather than the diagnostic capability achieved in contemporary specialist settings.

From an imaging perspective, the #ENZIAN classification has limitations, as it was originally developed as a surgical staging system and retrospective application to ultrasound reports can be challenging. However, #ENZIAN was selected as a retrospective analytical framework because it enabled consistent anatomical mapping of historical ultrasound and operative reports within a common classification system. Although the IDEA consensus provides a structured protocol for ultrasound acquisition and reporting, it is not designed as a cross‐modal classification framework. As many examinations within this cohort pre‐dated widespread implementation of IDEA‐based protocols, imaging practice and report structure were variable and not standardised according to contemporary guidelines. Consequently, #ENZIAN provided the most practical method for harmonising retrospective free‐text reports and facilitating direct comparison between preoperative eTVUS findings and laparoscopic disease distribution. Therefore, the findings should be interpreted within the context of early implementation of eTVUS, during which structured scanning protocols, systematic compartment‐based assessment, and operator pattern‐recognition skills were still evolving. With increasing adoption of IDEA‐based protocols and standardised endometriosis imaging pathways, further improvements in diagnostic performance, reporting consistency, and imaging–surgical concordance would be expected.

### 4.1 Limitations

This study has several imaging‐related limitations. The retrospective design relied on existing ultrasound and operative reports, which varied in detail and consistency, limiting the precision of retrospective #ENZIAN classification. Imaging and surgical classifications were completed by different clinicians and at different times, introducing the potential for interpretive bias. Formal assessment of inter‐observer variability was not feasible due to the retrospective design and reliance on a single clinical report per examination. The application of #ENZIAN within the developing IDEA‐based imaging protocol also presents structural challenges, as several #ENZIAN compartments, including parametrial, uterosacral, and rectovaginal disease, are not fully aligned with standard IDEA assessment steps, potentially leading to under‐representation of these regions in routine imaging reports.

Translation of free‐text imaging and operative descriptions into #ENZIAN categories required interpretation, which may introduce coding variability despite the use of pre‐specified rules and secondary review. Although complex or uncertain cases were escalated to senior reviewers to improve accuracy, the subjectivity inherent in retrospective morphological classification cannot be fully eliminated.

Finally, the study period spanned an era of evolving eTVUS protocols and increasing operator familiarity with deep endometriosis assessment. As such, variations in technique, confidence, and pattern‐recognition skills during early protocol development may have contributed to inconsistency in imaging performance and reporting quality. Furthermore, the study did not include a comparator cohort of patients assessed using routine pelvic ultrasound protocols prior to the implementation of eTVUS. As a result, although the findings provide insight into diagnostic performance during an early phase of eTVUS adoption, they do not permit direct evaluation of whether eTVUS performed better than preceding ultrasound approaches or quantify the magnitude of any improvement over time.

## 5 Conclusion

This study highlights the need for systematic scanning protocols, including a compartment‐based approach, advanced operator training, and structured reporting, extending beyond the historic protocols of a pelvic ultrasound practice. Such an approach moves beyond past pelvic ultrasound practice by ensuring that all relevant anatomical sites are consistently interrogated for deep, ovarian, peritoneal, and adhesional disease. These lessons should guide future protocol development and research. Prospective studies incorporating uniform templates, predefined training standards, and integration with complementary imaging such as MRI will be key to refining diagnostic strategies and sustaining progress.

## Funding

No external funding was provided for this research.

## Conflicts of Interest

The authors declare no conflicts of interest.

## Data Availability

The data that support the findings of this study are available on request from the corresponding author. The data are not publicly available due to privacy or ethical restrictions.
